# Coronary artery flow velocity reserve during the cold pressor test in overweight, healthy women using spiral imaging at 3 T

**DOI:** 10.1186/1532-429X-11-S1-O42

**Published:** 2009-01-28

**Authors:** Alice Y Chang, Melanie Kotys, Ivan Dimitrov, Andrew Kontak, Hardik Yadav, Christopher Maroules, Tommy Tillery, Ron Peshock

**Affiliations:** 1 UT Southwestern Medical Center at Dallas, Dallas, TX USA; 2 Philips Medical Systems, Cleveland, OH USA; 3 University of Texas School of Public Health, Dallas, TX USA

**Keywords:** Right Coronary Artery, Cold Pressor Test, Coronary Flow Velocity Reserve, Coronary Flow Velocity, Coronary Artery Flow

## Introduction

Women with chest pain demonstrate coronary endothelial dysfunction before obstructive disease can be appreciated by angiography. Invasive measurements of coronary artery vasoreactivity have been shown to predict future cardiovascular events. However, there are significant risks associated with invasive studies. Thus, the ability to non-invasively assess coronary vasoreactivity would be especially useful in the early diagnosis and management of women with coronary artery disease.

Changes in coronary flow velocity have been successfully measured by magnetic resonance flow mapping in response to handgrip stress at 3 T. The challenges for imaging women at risk for heart disease include the (1) smaller size of their arteries and (2) the high prevalence of overweight or obesity. In addition, we tested whether cold pressor stress, another widely used test of endothelial function that elicits a remarkable central sympathetic response, could provoke greater changes in coronary flow.

## Purpose

We sought to determine the feasibility of coronary flow velocity measurements in response to the cold pressor test in overweight women using 3 T MRI.

## Methods

Healthy, pre-menopausal women were recruited for this study and provided informed consent approved by the institutional review board. Subjects were placed supine in a 3 T MRI scanner (Achieva, Philips, Best, NL) using a 6 element cardiac receive coil. Scout scans were performed to determine the imaging plane orthogonal to the proximal right coronary artery (RCA). Baseline coronary velocity measurements were obtained using a VCG triggered breath-hold (10 to 12 seconds) velocity-encoded spiral cine sequence perpendicular to the RCA (FOV 256 × 256 mm^2^, matrix = 312 × 312, spatial resolution = 0.8 × 0.8 × 7 mm^3^, TR = 34 ms, TE = 3.5 ms, RF excitation angle = 20°, spiral interleaves = 11, VENC = 35 cm/s, temporal resolution = 69 ms). Heart rate and blood pressure were measured every 30 seconds during baseline imaging, stress and into recovery. After baseline imaging, the subject's left hand was placed in a half-water, half-ice bath for 3 minutes. Two successive velocity-encoded images of the RCA were acquired during the CPT. After the subject's hand was removed from the water, images were acquired at 1, 2 and 10 minutes into recovery. Images were analyzed using Q Flow (version 4.1.6, Medis, Leesburg, Virginia), and peak diastolic coronary velocity was determined as the maximum velocity during the diastolic rest period. Coronary flow velocity reserve was calculated as peak diastolic velocity during stress divided by baseline velocity. Statistical analysis was performed using a one-way analysis of variance (ANOVA) with Tukey post-hoc tests of significance between specific time points.

## Results

**Figure 1:**
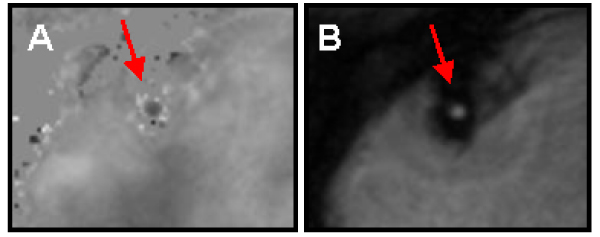
**Cross-sectional images of the RCA at rest in a subject using velocity-encoded MRI**. (A) Phase image and accompanying (B) Magnitude image.

**Figure 2:**
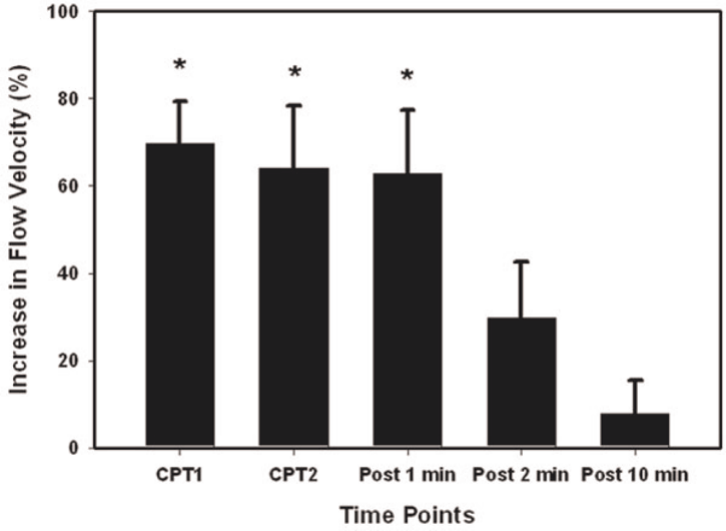
**Percent changes in peak diastolic flow velocity during cold pressor test**. *p < 0.01 compared to baseline and 10 minutes recovery.

The mean age of the subjects (n = 7) was 35.1 ± 6.5 (mean ± SD) and mean body mass index was 25.1 ± 1.8. Rate-pressure product increased 46.0 ± 23.0% from baseline to peak effect during CPT. Peak diastolic velocity increased significantly from baseline (13.3 ± 3.9 cm/s) to both CPT time points (22.7 ± 7.3 cm/s and 21.6 ± 7.5 cm/s) and one minute into recovery (21.8 ± 8.6 cm/s) (each p < 0.01). Recovery to baseline was achieved by 10 minutes. The average increase in peak diastolic flow velocity from baseline to peak cold pressor effect was 82.7 ± 29.2%. The mean coronary flow velocity reserve was 1.83 ± 0.29. Figures 1 and 2.

## Conclusion

Cold pressor test provokes a significant increase in coronary flow velocity that is measurable at 3 T in overweight women. Compared to previous reports of hand-grip stress during MRI, the CPT stimulated a greater increase in rate-pressure product (46% CPT v. 25.0% hand-grip) and coronary flow velocity (82.7% CPT v. 39.6% hand-grip) which persists one minute after withdrawal of the stress. Future studies will examine if this greater potential for stress-provoked changes can better detect more subtle differences in cardiovascular risk factors or responses to treatments.

